# ^47^Sc production development by cyclotron irradiation of ^48^Ca

**DOI:** 10.1007/s10967-017-5321-z

**Published:** 2017-06-13

**Authors:** R. Misiak, R. Walczak, B. Wąs, M. Bartyzel, J. W. Mietelski, A. Bilewicz

**Affiliations:** 1 Institute of Nuclear Physics, Polish Academy of Sciences, Radzikowskiego 152, 31-342 Cracow, Poland; 2 Institute of Nuclear Chemistry and Technology, Dorodna 16, 03-195 Warsaw, Poland

**Keywords:** Scandium radionuclides, Radionuclide therapy, Radioisotope production

## Abstract

The therapeutic radionuclide ^47^Sc was produced through the ^48^Ca(p,2n) channel on a proton beam accelerator. The obtained results show that the optimum proton energies are in the range of 24–17 MeV, giving the possibility to produce ^47^Sc radionuclide containing 7.4% of ^48^Sc. After activation, the powdery CaCO_3_ target material was dissolved in HCl and scandium isotopes were isolated from the targets. The performed separation experiments indicate that, due to the simplicity of the operations and the chemical purity of the obtained ^47^Sc the best separation process is when scandium radioisotopes are separated on the 0.2 µm filter.

## Introduction

Only few pairs of radionuclides have been proposed for theranostics applications. These are iodine radioisotopes ^123^I and ^124^I for imaging, as well as ^131^I for therapy. Other examples are ^86^Y, ^61^Cu and ^64^Cu for PET imaging and β^−^ emitters ^90^Y and ^67^Cu for therapy. Recently, scandium radioisotopes were also proposed for theranostics application. Significant attention in ^44^Sc and ^43^Sc as tracers in positron emission tomography imaging has been observed. ^44^Sc was first proposed by Rösch as a potential alternative for ^68^Ga in clinical PET diagnosis [1, 2]. ^44^Sc decays by the emission of low-energy positrons (*E*_β+_ = 1.47 MeV) with the half-life *T*_1/2_ = 3.97 h which is almost four-fold longer than that of ^68^Ga. ^44^Sc can be obtained from the ^44^Ti/^44^Sc generator [3] or produced in the ^44^Ca(p,n)^44^Sc reaction on small or medium medical cyclotrons that currently supply ^18^F to hospitals [4–9]. However, the co-emission of high-energy γ-rays (*E*_γ_ = 1157, 1499 keV), has to be taken into consideration with regard to radiation dose to the patients and clinical staff. Emission of high-energy γ-rays generates also radiolytic decomposition of biomolecules, which is thought to be accompanied by formation of free radicals [10]. Another radionuclide of scandium-^43^Sc, shows properties similar to ^44^Sc, but emits much lower energy of concurrent gamma. ^43^Sc can be produced either by the ^43^Ca(p,n) [11], or by ^42^Ca(d,n) [12] reaction, but unfortunately the cost of enriched calcium targets is very expensive. The more promising method of ^43^Sc production is alpha irradiation of natural calcium target through the ^40^Ca(α,p) and ^40^Ca(α,n) channels has been mentioned in recently published paper [13].

Another scandium radioisotope, i.e., ^47^Sc (*T*_1/2_ = 3.4 days, *E*_β-(av)_ = 162 keV, main *E*_γ_ = 159.4 keV, *I* = 68.3%) is a promising low-energy β^−^ emitter for targeted radiotherapy [14–17], thereby the β^+^ emitting radionuclides ^44^Sc or ^43^Sc together with the β^−^ emitting ^47^Sc represent ideal theranostic pairs as mentioned above regarding iodine, copper and yttrium radioisotopes.

The method of producing highly active ^47^Sc in a nuclear reactor was described by Mausner et al., Kolsky et al. and Srivastava et al. [18–20]. An enriched ^47^TiO_2_ target was irradiated with high energy neutrons (*E*_n_ > 1 MeV) to produce ^47^Sc via the ^47^Ti(n, p)^47^Sc reaction. The second method of ^47^Sc production is thermal neutron irradiation of ^46^Ca enriched target in the ^46^Ca(n,γ) → ^47^Ca(*T*_1/2_ = 4.54 days) (β^−^) → ^47^Sc nuclear reaction. This method was described recently by Muller et al. [21]. The former nuclear reaction requires *E*_n_ > 1 MeV, while the latter reaction uses more available thermal neutrons. The second advantage of this method is the use of ^47^Ca*/* ^47^Sc generator system to supply ^47^Sc activity, but the disadvantage is the requirement of an enriched target. ^46^Ca is presently available with only a 30% enrichment and at a very high price, which makes the target cost prohibitive.

Other ways of producing ^47^Sc have been based on proton irradiation of ^nat^Ti [22] and γ irradiation of ^48^Ti in an electron linear accelerator (LINAC) [23]. The production efficiency was lower than in the former cases. Both production routes require also radiochemical separation of ^47^Sc from the Ti targets. Recently, new cyclotron method for ^47^Sc production by alpha irradiation of ^44^Ca enriched target was reported [24]. Unfortunately due to low cross section of the ^44^Ca(α,p)^47^Sc reaction production by this method makes difficult to obtain GBq quantities, which are necessary to carry out clinical trials.

Various methods of ^47^Sc separation from TiO_2_ targets based on tributyl phosphate (TBP) extraction, extraction chromatography or on cation and anion exchange processes have been reported [11, 12]. Dissolution in hot concentrated H_2_SO_4_ and evaporation of the solution were the most difficult and time-consuming steps in the case of the TiO_2_ target, what significantly limits the use of this type of targets. In the case of calcium targets many methods of scandium radionuclide separation have been reported [4, 5, 8, 22]. All proposed methods are simple, fast and allow for high percent of scandium radionuclides and for calcium recovery.

In the present work we propose an alternative way of ^47^Sc production through the ^48^Ca(p,2n) reaction at medium size cyclotrons (proton energy below 30 MeV). During ^48^Ca irradiation the ^48^Sc (*T*_1/2_ = 43.67 h) and ^46^Sc (*T*_1/2_ = 83.79 days) are also co-produced, therefore the goal of our studies was optimization of the parameters of ^48^Ca irradiation for maximization the ^47^Sc production with minimal ^48^Sc and ^46^Sc impurities.

## Experimental

### Materials and methods

Hydrochloric acid (HCl), 37%, glacial acetic acid (CH_3_COOH) and citric acid were purchased from Sigma-Aldrich, sodium hydroxide (NaOH) from Merck, sodium acetate from POCH S.A. (Gliwice, Poland), ammonia and ethanol from Fluka. All chemicals were analytical grade and were used without further purification. Chelating ion exchange resin Chelex 100, (Na^+^-form, mesh size 100–200, bed size 0.8 × 4.0 cm, suspended in water) was purchased from Bio-Rad Laboratories.

### Irradiation of calcium target

Production yield of ^47^Sc and ^48^Sc radionuclides for proton induced reactions on CaCO_3_ with natural isotopic composition were measured as function of proton energy in the range of 60 → 0 MeV using activation method on stacks. The stack consisted of thin metallic foils with natural isotopic composition interleaved with CaCO_3_ targets. The stack was assembled from six groups of Al–Cu–CaCO_3_ and five groups of Al–Cu–Ti–CaCO_3_. The stack of targets and foils were mounted in a target holder which was made of aluminum. CaCO_3_ powder of analytical grade from POCH S.A. (Gliwice, Poland) were pressed with 232 MPa. Thickness of targets were about 0.35 g/cm^2^. The thickness of monitor reaction Al, Cu and Ti foils (purity 99.0–99.9% supplied by Goodfellow Cambridge Ltd., England) was 0.02 mm for Al, 0.01 mm for Cu and 0.01 mm for Ti. The thin metallic foils were used to monitor intensity and/or energy of proton beam.

Two stacks were irradiated at the extracted proton beam of the AIC-144 cyclotron of the Institute of Nuclear Physics Polish Academy of Sciences Cracow. Irradiations were carried out for 5 h with beam current of about 30 nA and with initial proton bombarding energy of 60 MeV.

### Data analysis

The activities of the radioactive products of the targets and monitors were measured nondestructively using the gamma HPGe-detector coupled with Multichannel Analyzers 919E EtherNIM. The photo peak area of γ-ray spectra was determined by using MAESTRO Multichannel Analyzer Emulation. The decay data for the monitors and Sc radionuclides, such as half-life (*T*_1/2_), γ-ray energy (*E*_γ_) and γ-ray emission probability (*I*_γ_), were taken from the Table of Radioactive Isotopes [25].

The proton flux intensity was determined through the monitor reactions: ^27^Al(p,x)^22,24^Na, ^nat^Cu(p,x)^56^Co, ^62,65^Zn and ^nat^Ti(p,x)^48^V from the measured activities induced in monitor foils at the front position of each CaCO_3_ target. The standard cross-sections for the monitor reactions were taken from [26].

The energy degradation along the stacks and effective particle energy in the middle of each foil was calculated using the computer program SRIM version 2008.04 [27]. The estimated uncertainty of the points representing the proton energy ranges from ±0.6 up to ±1.5 MeV.

The following sources of errors were considered to derive the summed up uncertainty in the yield values of ^46^Sc, ^47^Sc and ^48^Sc: statistical error (1–7%), error of the monitor flux (~6%), error due to the sample thickness determination (1–2.5%) and error of efficiency calibration of γ-rays spectrometer (~5%). The overall uncertainty in the determined yield was around 12%.

### Separation of Sc from the target

Three methods previously elaborated for ^43,44^Sc production were tested for separation of ^47^Sc from natural Ca target. First of the methods based on application of chelating resin, elaborated in our group [4], consisted of dissolution of the target in 1 M HCl and adsorption of scandium radionuclides on chelating ion exchange resin Chelex 100 of bed size 0.8 × 4.0 cm and conditioned with 5 ml of 1 M HCl. After adsorption of Sc^3+^ and Ca^2+^, the column was washed with 30 ml of 0.01 M HCl in order to remove Ca^2+^. The scandium radionuclides were then eluted with 1 M HCl in 0.5 ml fractions. In the second method, described by Valdovinos et al. [8], the irradiated ^nat^CaCO_3_ target was dissolved in 1 ml of 9 M HCl solution. The dissolved target solution was passed through a column containing 50 mg of UTEVA resin and after adsorption of scandium radionuclides the column was washed with 5 ml of 9 M HCl. The scandium radionuclides were eluted with a 400 µl portion of H_2_O. The third method which used ^47^Sc separation on 0.2 µm filter was recently described by Minegishi et al. [24]. In this method calcium target was dissolved in 0.5 M HCl and next was neutralized by 25% NH_3_ solution to pH 10. The obtained Sc solution was then passed through a 0.2 μm filter (Whatmann) to trap Sc radioisotopes. Subsequently, 3 ml of pure water was passed through the filter to wash out residual Ca^2+^ and NH_4_^+^ cation. Scandium radionuclides trapped on the filter was eluted by 0.5 M HCl. The methods were tested on proton irradiated natural Ca targets containing ^44^Sc, ^47^Sc and ^48^Sc no carrier added radionuclides.

## Results and discussion

Up to now for the production of ^46,47,48^Sc through the ^nat^Ca(p,xn)^46,47,48^Sc reaction only two groups reported experimental data: first group reported cross section data using ^nat^Ca in calcium formate as the target [28] and a second group a thick target yields for natural calcium metal [6]. The first paper published by Michel et al. describes cross section data over the proton energy only in the range 70 → 24 MeV for ^nat^Ca(p,xn)^46^Sc and in the proton energy range 70 → 15 MeV for ^nat^Ca(p,xn)^48^Sc. The second paper published by Severin et al. which described result of the ^44^Sc production on natural calcium target, contains also results of production ^47^Sc and ^48^Sc contaminants at 16 MeV proton energy. So far there are no systematic studies on ^47^Sc production in ^48^Ca(p,2n)^47^Sc reaction. In the present work results of ^47^Sc production via the ^48^Ca(p,2n) reaction in energy range 60–0 MeV have been described.

Due to low availability and high cost of ^48^Ca we decided to work with natural calcium targets containing 0.187% ^48^Ca. Table 1 shows the isotopic compositions of the natural and ^48^Ca enriched calcium targets.

**Table 1:** Isotopic composition of natural abundance and of enriched calcium targets (Sigma-Aldrich and ISOFLEX, USA)

	^40^Ca (%)	^42^Ca (%)	^43^Ca (%)	^44^Ca (%)	^46^Ca (%)	^48^Ca (%)
^nat^Ca	96.94	0.647	0.135	2.086	0.004	0.187
^48^Ca	27.9	0.3	0.1	2.2	<0.1	69.2

Table 2 shows results of irradiation of ^nat^CaCO_3_ in the energy range 60–0 MeV.

**Table 2:** Measured production yields of the reactions ^nat^Ca(p,xn)^46,47,48^Sc at EOB as a function of proton bombardment energy

Energy interval (MeV)	Average energy (MeV)	Measured yield (kBq/μA h)			
		^46^Sc	^47^Sc	^48^Sc	^47^Ca
60.0–56.5	58.2 ± 0.6	0.46 ± 0.06	14.49 ± 1.59	6.93 ± 0.76	22.82 ± 1.53
56.1–52.7	54.4 ± 0.6	0.52 ± 0.053	16.12 ± 1.61	7.33 ± 0.77	22.34 ± 1.61
52.4–48.8	50.6 ± 0.6	0.64 ± 0.07	16.08 ± 1.90	7.48 ± 0.72	23.02 ± 1.29
48.4–44.6	46.5 ± 0.6	0.84 ± 0.1	18.21 ± 1.64	8.35 ± 0.69	22.10 ± 1.28
44.2–40.2	42.1 ± 0.8	1.15 ± 0.13	20.83 ± 1.46	9.22 ± 0.75	23.70 ± 1.44
39.7–36.2	37.9 ± 0.8	2.09 ± 0.21	28.43 ± 3.41	11.01 ± 0.91	24.53 ± 1.45
35.7–31.2	33.4 ± 1.0	2.51 ± 0.23	38.09 ± 3.43	10.55 ± 0.86	24.44 ± 1.17
30.6–24.8	27.7 ± 1.2	1.84 ± 0.17	66.10 ± 5.29	13.68 ± 1.08	25.06 ± 1,37
24.1–16.9	20.5 ± 1.3	0.35 ± 0.04	143.95 ± 9.36	21.17 ± 1.57	20.10 ± 0.92
16.6–5.7	11.1 ± 1.5	0.075 ± 0.009	84.63 ± 6.77	100.84 ± 6.86	4.61 ± 0.35
5.3–0	2.6 ± 1.5	0.03 ± 0.004	2.55 ± 0.30	22.24 ± 1.96	0.69 ± 0.07

As shown in Table 2 the optimum energy range for the ^48^Ca(p, 2n)^47^Sc reaction is 24 → 17 MeV with the peak at about 20 MeV for CaCO_3_ target. The peak for ^48^Ca(p,n)^48^Sc reaction is about 11 MeV and is about 33 MeV for ^48^Ca(p,3n)^46^Sc reaction. It should be noted that ^46^Sc forms also in ^46^Ca(p,n)^46^Sc nuclear reaction, but due to the very low abundance of ^46^Ca in ^nat^Ca (0.004%) the produced ^46^Sc activity is negligible. In the obtained ^47^Sc radionuclide the percent of ^48^Sc impurity at EOB is 14.7 and decreases after 80.38 h (half-life of ^47^Sc) to 7.4%. As shown in Table 2 in this energy range also 20.1 kBq/μA h of ^47^Ca (*T*_1/2_ = 4.5 days) is produced which by the β^−^ decay generates additional ^47^Sc activity, therefore the yield of ^47^Sc at EOB includes also the activity of ^47^Sc generated from ^47^Ca decay during the irradiation. The ratio of ^46^Sc impurity to ^47^Sc was only 0.2% at EOB. Irradiation of thiner CaCO_3_ targets could allow to increase the representing points in the proton energy range.

The yields estimated by using the cross section data from Michel et al. [28] are lower by about 25% than in our work. Irradiations of natural Ca metal at 16 MeV proton energy were performed by Severin et al. [6]. This group presented yields at EOB equal to 0.09 for ^47^Sc and 0.33 MBq/μA h for ^48^Sc. However, direct comparison of obtained experimental yield values is difficult because irradiated Ca targets had different chemical forms. For comparison of measured yields obtained in this work with data obtained by Severin et al. [6], a normalization for the number of Ca atoms in the target was performed. Our yield value 0.31 MBq/μA h for ^48^Sc, after the normalization, over the proton energy range 16.6 → 0 MeV is in good agreement with the yield value obtained by Severin et al. [6]. In the case of ^47^Sc we obtained about two times higher yield value than Severin et al. [6]. This difference may result from error in determination of input proton energy on the target. In this proton energy range a small change in energy causes a large change in the efficiency of (p,2n) nuclear reaction.

To find the best method for separation of ^47^Sc from the calcium targets three methods previously elaborated for ^44^Sc were tested. These methods were compared in respect to Sc separation yield, possibility of separation from other metallic impurities which could negatively affect the effectiveness of ^47^Sc bioconjugates labeling and recovery of calcium target (Table 3).

**Table 3:** Comparison of the methods for separation of scandium radionuclides from calcium targets

Separation method	Eluent of Ca	Eluent of Sc	Recovery Sc (%)	Recovery CaCO_3_ (%)
Chelex-100 chelating resin	0.01 M HCl	0.1 M HCl	85	87
UTEVA extraction resin	9 M HCl	H_2_O	79	90
Filtration (0.2 µm)	25% NH_3aq_	0.5 M HCl	96	93

All separation procedures studied are fast and simple. In the case of Chelex 100 and UTEVA resins the target dissolution and separation of scandium radioisotopes were performed in 30 min and the separation process on 0.2 µm filter needs only 15 min. The separation process on 0.2 µm filter gives also highest recovery of scandium isotopes and is much simpler and faster in comparison with other methods studied. It contains only two simple processes: adsorption Sc(OH)_3_ on the filter and then dissolution of the precipitate in hydrochloric acid.

Chemical purity of the Sc product is important, since the presence of other metals may interact with the DOTA-chelator. The most dangerous is Fe^3+^ for which forms stronger complexes with DOTA than Sc^3+^ [29]. Influence of other possible impurities like Zn^2+^, Mg^2+^, Sr^2+^ and Co^2+^ is negligible due to the much lower stability constants of their DOTA complexes [29]. The results of Fe concentration in dissolved calcium targets and in scandium fractions after separation processes are presented in Table 4.

**Table 4:** Iron concentration in dissolved calcium target in HCl solutions and in scandium fractions after separation processes

Separation methods	Concentration HCl for target dissolution [M]	Fe concentration in dissolved target (ppm)	Fe concentration scandium fractions (ppm)
Chelex-100 chelating resin	1	1.54	0.99
UTEVA extraction resin	9	7.7	<0.001
Filtration (0.2 µm)	0.5	28.8	0.07

For all the methods studied the concentration of Ca^2+^ in Sc fractions was less than 1 ppm.

Taking into account the obtained results, we recommend filtration process for the separation of Sc radionuclides from the calcium targets.

## Conclusion

The production of ^47^Sc in (p,2n) nuclear reaction on natural CaCO_3_ target was successfully performed. During proton irradiation of natural calcium target radionuclides of interest ^46^Sc, ^47^Sc and ^48^Sc can be formed only in p,n, p,2n and p,3n reactions on ^48^Ca, therefore, the results obtained for natural calcium can be recalculated for enriched ^48^Ca targets. The obtained results of production efficiency creates the opportunity to produce GBq activity levels of ^47^Sc. The separation process based on precipitation of Sc(OH)_3_ and separation on 0.2 µm filter is simple, reliable, efficient and fast and can be easily adapted for remote operation.
